# Pulmonary Artery Filling Defects in COVID-19 Patients Revealed Using CT Pulmonary Angiography: A Predictable Complication?

**DOI:** 10.1155/2021/8851736

**Published:** 2021-03-10

**Authors:** Arnaldo Scardapane, Laura Villani, Davide Fiore Bavaro, Francesca Passerini, Amato Antonio Stabile Ianora, Nicola Maria Lucarelli, Gioacchino Angarano, Piero Portincasa, Vincenzo Ostilio Palmieri, Annalisa Saracino

**Affiliations:** ^1^Interdisciplinary Department of Medicine-Section of Diagnostic Imaging, University Hospital “Policlinico” of Bari Medical School, Italy; ^2^Department of Biomedical Sciences and Human Oncology, Clinic of Infectious Diseases, University Hospital “Policlinico” of Bari Medical School, Italy; ^3^Department of Biomedical Sciences and Human Oncology-Division of Internal Medicine “Augusto Murri”, University Hospital “Policlinico” of Bari Medical School, Italy

## Abstract

**Purpose:**

This study is aimed at assessing the prevalence of pulmonary artery filling defects (PAFDs) consistent with pulmonary artery embolism (PAE) in patients with SARS-CoV-2 infection and at investigating possible radiological or clinical predictors.

**Materials and Methods:**

Computed Tomography Pulmonary Angiographies (CTPAs) from 43 consecutive patients with a confirmed COVID-19 infection were retrospectively reviewed, taking into consideration the revised Geneva score and the D-dimer value for each patient. Filling defects within the pulmonary arteries were recorded along with pleural and parenchymal findings such as ground glass opacities, consolidation, crazy paving, linear consolidation, and pleural effusion. All these variables were compared between patients with and without PAFD. The predictive performance of statistically different parameters was investigated using the receiver operating characteristics (ROC).

**Results:**

Pulmonary embolism was diagnosed in 15/43 patients (35%), whereas CTPA and parenchymal changes related to pulmonary COVID-19 disease were evident in 39/43 patients (91%). The revised Geneva score and the mean D-dimer value obtained using two consecutive measurements were significantly higher in patients with PAFD. The ROC analysis demonstrated that a mean D-dimer value is the parameter with the higher predictivity (AUC 0.831) that is a cut‐off value > 1800 *μ*g/l which predicts the probability of PAFD with a sensitivity and specificity of 70% and 78%, respectively.

**Conclusions:**

This single centre retrospective report shows a high prevalence of pulmonary artery filling defects revealed using CTPA in COVID-19 patients and demonstrates that the mean value of multiple D-dimer measurements may represent a predicting factor of this complication.

## 1. Introduction

COVID-19 pneumonia, caused by the virus SARS-CoV-2 (severe acute respiratory syndrome coronavirus-2), was first reported in China in December 2019 and spread rapidly in many countries until it was declared a pandemic by the WHO on 11th March 2020 [1]. SARS-CoV-2 infection is highly contagious and is transmitted from human-to-human through respiratory droplets and contact [2]. The main symptoms are fever, dry cough, fatigue, and malaise. In the advanced stage of the disease, patients may develop dyspnoea and respiratory distress syndrome (ARDS). However, it is the heterogeneity of the symptoms which poses a serious challenge to the healthcare providers as regards the most appropriate clinical management [3].

Computed Tomography (CT) plays an important role in the diagnosis and follow-up of COVID-19 pneumonia, thus allowing the best clinical management. Current guidelines advocate the use of the high resolution unenhanced chest CT (HRCT) in COVID-19 patients thanks to its elevated accuracy in detecting pulmonary changes in viral pneumonia [3, 4], also permitting differential diagnoses [5–7].

According to the literature, repeated CT follow-ups in COVID-19 patients demonstrate the transition from pure ground glass opacities (GGO) to GGO with consolidation as the disease's most frequent course [8, 9]. Reports of acute pulmonary artery embolism (PAE) associated with COVID-19 have emerged in the literature since the onset of the disease [10–13]. The hypothetical pathogenesis for SARS-CoV-2-induced thrombosis includes a disease-specific hypercoagulable state, diffused cytokine-mediated microvascular damage, and, in a few cases, reactive thrombocytosis [14, 15].

Indeed, due to its elevated accuracy in detecting the embolic filling defects in the pulmonary arteries, CT Pulmonary Angiography (CTPA) has become fundamental in COVID-19 patients with suspected PAE [2, 3, 16, 17].

The purpose of our study was to assess the role of CTPA in the detection of pulmonary artery filling defect (PAFD) consistent with PAE and to investigate possible radiological or clinical predictors of PAFD in a cohort of patients with COVID-19 infection in a “COVID hospital” in southern Italy during the pandemic.

## 2. Materials and Methods

### 2.1. Patients

The study is a retrospective evaluation of all COVID-19 patients who received a CTPA in the period between 1st March 2020 and 30th April 2020. For this purpose, 118 CT scans of COVID-19 patients were retrieved using our RIS/PACS system (Fenix Elco Health Systems/Carestream Health, Rochester, NY) from the COVID department of the University Hospital “Policlinico” of Bari (Italy). 75/118 (64%) unenhanced scans were excluded. The remaining 43/118 (36%) patients (22 men and 21 women, mean age 65 years) satisfying our inclusion criteria, being in possession of a CT Pulmonary Angiography, were retained for the study (clinical data are summarized in Table 1).

The local ethical committee was informed and approved the study; all patients signed an informed consent which included their enrolment in eventual retrospective studies.

All patients tested positive to SARS-CoV-2 following a RT-PCR test on nasopharyngeal sampling.

CTPA was carried out in cases of clinical suspicion of PAE. No CTPAs were performed on patients on mechanical ventilation in the intensive care unit (ICU).

All CT examinations were acquired with a 128-row multidetector CT scanner (Siemens SOMATOM Definition DS).

The CT chest examination consisted of an unenhanced high resolution CT scan (HRCT), followed by a CTPA after the intravenous administration of 50 ml of iodinated contrast agent (Iomeprol 400 mgI/ml) and of 25 ml of saline at a flow rate of 4 ml/s, through an antecubital vein, using an automatic power injector.

The bolus-tracking technique was used, with a threshold of 100 HU in the main pulmonary artery. Acquisition parameters were the following: slice thickness 0.6 mm, tube voltage 120 kVp, rotation time 0.33 s, pitch 1.2, and acquisition time 2.94 sec. Images were reconstructed with a slice thickness of 1 mm in mediastinal and lung settings.

The following clinical and laboratory parameters were recorded in tables: sex, age, BMI, fever, cough, thoracic pain, dyspnoea, smoking habits, days of hospitalization, enoxaparin treatment before CTPA, enoxaparin dosage, and need for oxygen therapy or noninvasive ventilation (NIV). For all patients, D-dimer measurement was available on the day of admission and within 24 h of the CT scan. The revised Geneva [18] score and Wells' score [19] were also retrospectively calculated based on the available clinical and laboratory parameters.

### 2.2. Image Analysis

CT scans were blindly reviewed on a PACS workstation in consensus by two radiologists with at least 15 years of experience. Filling defects within any branch of pulmonary arteries were recorded; the site of the PAFD was classified using a 4-point scale (adopted by the authors for the first time in this study), according to which branch was involved: 1 point for the main pulmonary artery, 2 for the lobar pulmonary artery, 3 for the segmental pulmonary artery, and 4 for the subsegmental pulmonary artery. In the event of multiple emboli, the one located in the more proximal vessel was recorded for statistical analysis. Each patient was then classified according to the number of lobes involved (1 to 5) and according to the prevalent pulmonary parenchymal pathological findings: pure ground glass opacities (GGO), prevalent consolidation, mixed GGO+consolidation, crazy paving pattern, and linear opacities. Pleural effusion and thoracic lymph nodes (>15 mm in the short axis) were also recorded.

### 2.3. Statistical Analysis

Continuous variables with normal distribution were compared using the Student *t*-test, while the nonparametric Mann–Whitney test was favoured when normal distribution was rejected. A comparison between categorical variables was performed using either the chi-squared test or Fisher's exact test. On the basis of CTPA, 15 categorical clinical or CT-based variables (sex, GGO, consolidation, crazy paving pattern, linear consolidation, pleural effusion, enlarged lymph nodes > 15 mm, fever, cough, thoracic pain, dyspnoea, smoking habits, need of oxygen therapy or NIV, and enoxaparin treatment before CTPA) and ten numeric variables (age, BMI, days of hospitalization before CTPA, number of affected lobes, revised Geneva score, and Wells' score D-dimer value at admission, D-dimer value within 24 h before CTPA, mean D-dimer value, and enoxaparin dosage) were considered. More precisely, the mean D-dimer value was calculated as follows: (D-dimer at admission + D-dimer within 24 h of CTPA)/2. The predictive capabilities of variables which resulted *significantly different* between patients with and without PAFDs were compared and graphed using ROC analysis assuming a binormal distribution [20]. Statistical analysis was performed using the STATA/IC version 14 software.

## 3. Results

The clinical, biological, and imaging features are summarized in Tables 1, 2, and 3.

**Figure 1:**
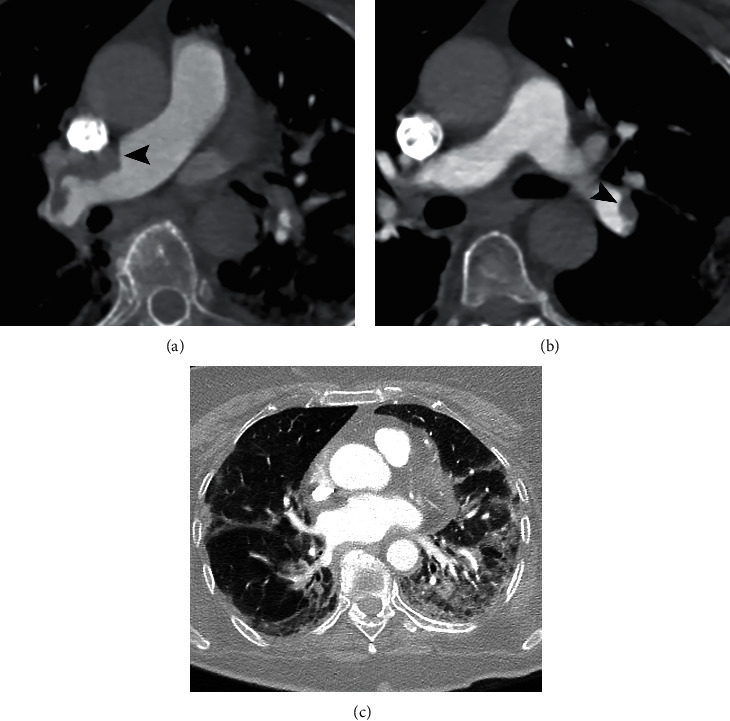
CTPA of an 81-year-old woman with COVID-19 disease and dyspnoea. (a) Mediastinal window shows PAFD defects in the main right pulmonary artery (arrowhead). (b) PAFDs are also recognized in the lobar artery for the left lower lobe (arrowhead). (c) Lung window shows bilateral GGO, consolidation, and linear opacities.

**Figure 2:**
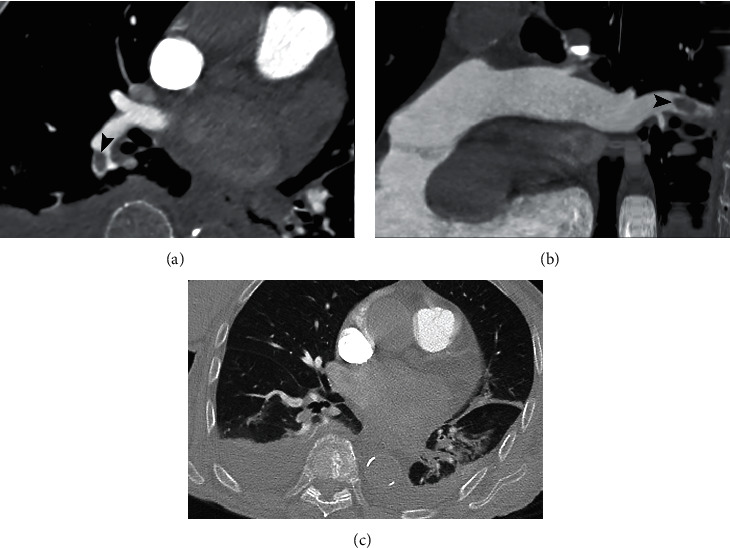
CTPA of a 94-year-old woman with SARS-CoV-2 infection. (a) Axial image with mediastinal window. (b) Curved MRP image with mediastinal window. PAFD of the lobar artery for the right lower lobe (arrowhead). (c) Lung window shows linear opacities and consolidation in the left lower lobe and bilateral pleural effusion.

**Figure 3:**
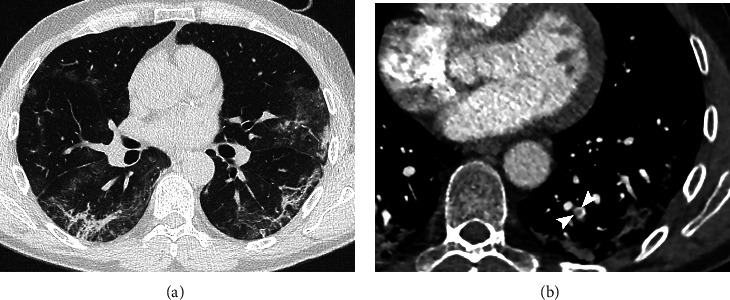
CTPA of a 58-year-old man with COVID-19 disease: (a) unenhanced HRCT shows multiple peripheral patchy GGO and bilateral linear opacities; (b) CTPA reveals an arterial filling defect in the subsegmental artery for left lower lobe (arrowheads).

PAFDs were diagnosed in 15/43 (35%) patients. PAFD was located in the main pulmonary artery in 1/15 (7%) patient (Figure 1), in 2/15 (13%) patients in lobar arteries (Figure 2), in 6/15 (40%) patients in segmental arteries, and in 6/15 (40%) in subsegmental branches (Figure 3). The number of cases receiving prophylactic enoxaparin therapy at the moment of CTPA as well as the dosage of enoxaparin was not significantly different between patients with and without PAFD.

Parenchymal pathological findings related to pulmonary COVID-19 disease were evident in 39/43 (91%) patients. In 32/43 patients (74%), more than 3 pulmonary lobes were involved. The number of patients with pure GGO or pure consolidation patterns was the same (15/43, 35%), while GGO+consolidation was detected in 7/43 (15%) patients (Figures 1 and 2). Crazy paving pattern and linear opacity were found, respectively, in 5/43 (12%) and 22/43 (51%) patients (Figure 3). Pleural effusion was diagnosed in 11/43 (26%) patients.

**Figure 4:**
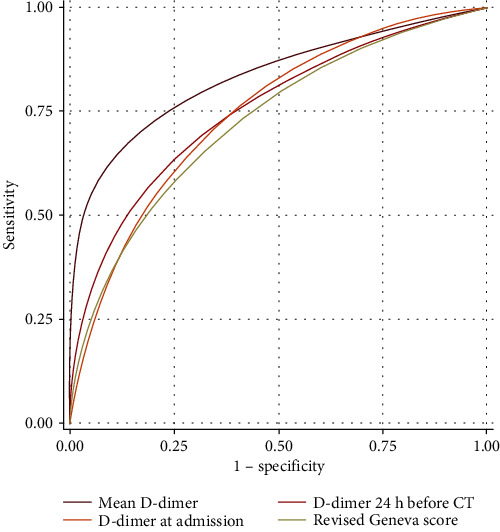
ROC curves comparing the predictivity of statistically significant variables.

D-dimer values in both measurements recorded (at admission and within 24 h of CTPA) and the mean value of the same were significantly higher in patients with PAE. Similarly, the revised Geneva score was higher in the patients with acute pulmonary embolus than in those without embolus (mean 2 ± 2 versus 4 ± 2, *p* = 0.011). No other statistically significant differences were found between patients with and without PAFD for all the remaining variables; namely, no differences were found in D-dimer values for patients with PAFD located in arterial branches of different grades according to an ANOVA test. ROC analysis demonstrated that the variable with a higher predictive value was the mean D-dimer value (AUC 0.831) and that a cut‐off value > 1800 *μ*g/l of this parameter predicted the probability of PAFD with a sensitivity and specificity of 70% and 78%, respectively (Table 4 and Figure 4).

## 4. Discussion

Our study demonstrated PAFD, consistent with PAE, in 15/43 (35%) consecutive CTPAs performed in COVID-19 patients; our results are in agreement with previous reports by Grillet et al. (a 23% prevalence of PAE on 100 CTPAs) and by Leonard-Lorant et al. (a 30% prevalence of PAE on 106 CTPAs) [2, 16]. This rate of PAE is higher than that of 3%-10% encountered in normal circumstances, when the ever-increasing availability of CTPA favours its inappropriate use, resulting in a low prevalence of PAE [21, 22]. The high prevalence of PAFD in our study is in agreement with other authors' hypothesis of a coagulopathy associated with COVID-19 infection and with the hypothesis that pulmonary vessel obstruction, detected by CTPA, may represent pulmonary thrombi rather than emboli, as they are not fully occlusive and are related to a low number of deep venous thromboses [23]. It is still unknown if this phenomenon is related to a direct endothelial cell involvement by the virus or to an inflammatory reaction following alveolar damage [24, 25]. This pathogenetic mechanism might also explain the overall lower predictivity of clinical scores, such as the Wells score, compared with recent papers [26]. Similar conclusions were reached by Poissy et al. who demonstrated a 22% prevalence of PAE in patients admitted to ICU during the pandemic period [27]. In our experience, no CT scans were performed in patients requiring intubation, who were mostly managed clinically, using pulmonary US for imaging. Consequently, our results confirm a higher prevalence of PAE even in less severe patients, strengthening the possibility of a disease-specific hypercoagulable condition. Most of the arterial filling defects found in our study were in segmental and subsegmental vessels. However, the severity of the pneumonia and the parenchymal CT findings was not significantly different in patients with or without PAE. Indeed, most of them showed a bilateral and multilobar involvement of the lungs. Subpleural consolidation, which was advocated as a parenchymal predictor of PAE, was not significantly associated with this complication [28]. In our study, BMI does not correlate with PAFD, in contrast with a recent paper by Poyiadji et al. [29]. The main reason for this discrepancy, in our opinion, is the high number of obese patients (BMI > 30) in Poyiadji et al.'s study, in comparison to the highest BMI of our patients which was 27.8. In agreement with previous literature, the D-dimer values at admission and within 24 h of CTPA, in addition to the mean value of these two measurements, were significantly higher in patients with PAE [2, 16, 29]. The high values of the D-dimer could be related to the activation of blood coagulation in COVID-19 patients following a systemic inflammatory response syndrome [23]. The revised Geneva scores were also significantly higher in COVID-19 patients showing PAE. ROC analysis showed that the mean D-dimer value was the variable with the highest predictivity in our study (AUC 0.831). These findings, in our opinion, are meaningful as they suggest that, though most of the patients have high D-dimer levels (often >1000 *μ*g/l), those with constantly higher levels may also show a higher risk for pulmonary artery obstruction. Our study has some limitations. First of all, it is a retrospective study from a single centre with a lower number of patients if compared with similar reports. Although Italy has been one of the most involved countries in the SARS-CoV-2 pandemia, southern regions have experienced a by far lower number of cases. Furthermore, in our centre, most patients were studied using an unenhanced chest CT while CTPA was performed on patients whose respiratory conditions were worsening. However, the selection criteria for CTPA were not established beforehand, and CTPA was obtained during the hospitalization (13 median days after admission) when the majority of the patients were already receiving prophylactic enoxaparin therapy. This data poses issues concerning the possible underestimation of PAE present at admission and the role of enoxaparin therapy in the management of this complication. Further investigation with prospective studies is required. Finally, in our study, no information is available regarding the ICU patients, as none of these underwent a CTPA. However, this drawback makes our experience different from other similar papers, as it underlines an increased prevalence of PAE unrelated to an admission to ICU, thus supporting the hypothesis of SARS-CoV-2-related endovascular thrombosis.

## 5. Conclusion

In conclusion, our results are in agreement with previous literature as it shows a higher risk of pulmonary artery obstruction in COVID-19 patients; our data suggest that patients with persistently high values of D-dimer should undergo CTPA, rather than HRCT, due to the higher risk of pulmonary artery obstruction. Nevertheless, further studies are needed to confirm our data and to explain the high prevalence of PAFD during SARS-CoV-2 infection.

## Figures and Tables

**Table 1:** Clinical features of 43 COVID-19 patients with CTPA.

Clinical features	Total (*N* = 43)	Absence of pulmonary embolism (*N* = 28)	Pulmonary embolism (*N* = 15)	*p* value
Mean age ± SD	65 ± 17	63 ± 16	69 ± 18	0.284
Sex M/F	22/21	16/12	6/9	0.289
BMI, median (IQR)	24.5 (22.3-27.8)	24.2 (22.2-28.1)	25.5 (22.4-27.1)	0.7
Days of hospitalization, median (IQR)	13.5 (10.6-21)	17 (11-26)	11 (4.8-21)	0.2
Signs and symptoms at the time of hospitalization, *n* (%)				
Fever	39 (91)	26 (93)	13 (87)	0.510
Cough	27 (63)	16 (57)	11 (73)	0.300
Dyspnoea	25 (58)	16 (57)	9 (60)	0.858
Thoracic pain	6 (14)	2 (7)	4 (27)	0.082
Laboratory tests, median (IQR)				
D-dimers hospital admission, *μ*g/l (v.n < 500)	1029 (598-1904)	775 (533-1158)	1650 (1319-4620)	0.002
D-dimers CTPA, *μ*g/l (v.n < 500)	1795 (1072-4451)	1370 (901-2182)	4000 (1647-9618)	0.009
Mean D-dimers	1412 (901-3738)	1131 (802-1746)	3719 (1798-6593)	0.001
Enoxaparin treatment before CTPA, *n* (%)	34 (80)	22 (79)	12 (80)	0.914
Enoxaparin dose	8400 (3400)	8700 (3430)	7555 (3570)	0.26
NIV, *n* (%)	18 (43)	14 (50)	4 (27)	0.144
Oxygen therapy, *n* (%)	17 (39)	11 (39)	6 (40)	0.964
Revised Geneva score, median (IQR)	3 (2-5)	2 (0-4)	4 (3-6)	0.013
Well's score, median (IQR)	1 (0-2)	1 (0-2)	2 (0-3)	0.170
Smoke, *n* (%)	3 (7)	1 (4)	2 (13)	0.237

**Table 2:** Radiologic findings of 43 COVID-19 patients with CTPA.

Radiologic findings	Total (*N* = 43)	Absence of pulmonary embolism (*N* = 28)	Pulmonary embolism (*N* = 15)	*p* value
CT features, *n* (%)				
Ground glass opacities	15 (35)	11 (39)	4 (27)	0.413
Consolidation	15 (35)	8 (29)	7 (47)	0.241
Ground glass opacities and consolidation	7 (16)	5 (18)	2 (13)	0.705
Crazy paving	5 (12)	4 (14)	1 (7)	0.463
Linear opacity	22 (51)	14 (50)	8 (53)	0.837
Lymphadenopathy	6 (14)	5 (18)	1 (7)	0.318
Pleural effusion	11 (26)	7 (25)	4 (27)	0.906
Number of affected lobes, *n* (%)				
0	4 (9.3%)	3 (11)	1 (7)	0.8
1	2 (4.7%)	2 (18)	0 (0)	
2	2 (4.7%)	1 (4)	1 (7)	
3	3 (7.0%)	0 (0)	3 (20)	
4	5 (11.6%)	2 (7)	3 (20)	
5	27 (62.8%)	20 (71)	7 (46)	

**Table 3:** Location of more proximal filling defect depictable on CTPA.

	No. of patients (%) (*N* = 15)
Main pulmonary artery	1 (7)
Lobar artery	2 (13)
Segmental artery	6 (40)
Subsegmental artery	6 (40)

**Table 4:** Multiple variable ROC comparison for predictors.

Variable	AUC	Std. err.	95% conf. interval
Mean D-dimer	0.831	0.084	0.666–0.996
D-dimer at admission	0.748	0.091	0.568–0.928
D-dimer within 24 h before CTPA	0.755	0.093	0.572–0.938
Revised Geneva score	0.727	0.103	0.525–0.929

## Data Availability

The data that support the findings of this study are available on request from the corresponding author, AS.

## Abbreviations

COVID-19: Coronavirus disease 2019
SARS-CoV-2: Severe acute respiratory syndrome coronavirus-2
ARDS: Acute respiratory distress syndrome
CT: Computed tomography
CTPA: CT-pulmonary angiography
HRCT: High resolution unenhanced chest CT
GGO: Ground glass opacities
PAE: Pulmonary artery embolism
PAFD: Pulmonary artery filling defect
ICU: Intensive care unit
ROC: Receiver operating characteristics.
